# Parotid sparing in RapidPlan Oropharynx models: To split or not to split

**DOI:** 10.1002/jmrs.376

**Published:** 2020-02-11

**Authors:** James O'Toole, Kenny Wu, Regina Bromley, Mark Stevens, Thomas Eade, Kirsten van Gysen, John Atyeo

**Affiliations:** ^1^ Department of Radiation Oncology Northern Sydney Cancer Centre Royal North Shore Hospital Reserve Road, St Leonards NSW Australia

**Keywords:** Head and Neck planning, knowledge‐based planning, Oropharynx model, parotid sparing, RapidPlan

## Abstract

**Introduction:**

Differences in knowledge and experience, patient anatomy and tumour location and manipulation of inverse planning objectives and priorities will lead to a variability in the quality of radiation planning. The aim of this study was to investigate whether parotid glands should be treated as separate or combined structures when using knowledge‐based planning (KBP) to create oropharyngeal plans, based on the dose they receive.

**Method:**

Two separate RapidPlan (RP) models were created using the same 70 radical oropharyngeal patients. The ‘separated model’ divided the parotids into ipsilateral and contralateral structures. The ‘combined model’ did not separate the parotids. The models were independently validated using 20 patients not included in the models. The same dose constraints and priorities were applied to planning target volumes (PTVs) and organs at risk (OARs) for all plans. An auto‐generated line objective and priority was applied in both models, with parotid mean dose and V50 doses evaluated and compared.

**Results:**

Plans optimised using the combined model resulted in lower ipsilateral mean doses and lower V50 doses in 80% and 75% of cases, respectively. Fifty‐five per cent of plans produced lower mean doses for the contralateral parotid when optimised using the combined model, while lower V50 doses were evenly split between the models.

**Conclusion:**

Combining the data for both parotids into one RP model resulted in better ipsilateral parotid sparing. Results also suggest that a combined parotid model will spare dose to the contralateral parotid; however, further investigation is required to confirm these results.

## Graphical abstract

Differences in knowledge and experience, patient anatomy and tumour location and manipulation of inverse planning objectives and priorities will lead to a variability in the quality of radiation planning. The aim of this study was to investigate whether parotid glands should be treated as separate or combined structures when using knowledge based planning (KBP) to create oropharyngeal plans. Combining the data for both parotids into one RP library resulted in better ipsilateral parotid sparing. Results also suggest that a combined parotid library will spare dose to the contralateral parotid, however further investigation is required to confirm these results.

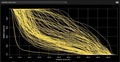

## Introduction

In the last decade, there has been a rise in the incidence of oropharyngeal carcinoma. This rise has been attributed to the increased incidence of human papilloma virus (HPV)‐related cancers.^1^, ^2^ HPV‐related cancers are highly responsive to treatment, with improved disease‐specific and overall survival.^3^ Combined chemoradiotherapy has become the preferred treatment option for these patients, with a 3‐year overall survival rate of 82.4%.^3^, ^4^ In view of their excellent response to treatment and prolonged survival, a major focus is on reducing treatment morbidity by improving treatment techniques and doses to organs at risk (OARs).

Previous studies have reported on significant improvements in survival as well as reduced toxicity in head and neck cancer patients treated with inversely planned radiation therapy.^4^, ^5^ However, the radiotherapy planning process for these complex head and neck treatments is intensive and can create workflow issues within departments. Differences in staff experience and knowledge lead to variability in plan quality, with more experienced staff having a higher success at manipulation of planning objectives and priorities based on individual patient tumour size and proximity to OARs.^6^, ^7^ Furthermore, randomised studies have also shown a benefit of standardised plan analysis to improve plan quality and patient outcomes.^8^ In an effort to address these challenges, there has been increasing interest in semi‐automated knowledge‐based planning (KBP) strategies. KBP tools such as RapidPlan™ (RP) have the potential to not only improve workflow but also improve plan quality.

Duke University^9^, ^10^, ^11^, ^12^, ^13^ and Washington University^14^, ^15^ were the early innovators of KBP, providing evidence of increased consistency and plan quality. With the use of recently developed commercial knowledge‐based software, others have since focused on the predictability and reliability of such strategies, and the number of patients needed in each model to establish optimal outcomes.^16^

RapidPlan™ is the commercial KBP software released by Varian Medical Systems (Palo Alto, USA). The software auto‐creates line objectives for various OARs based on data uploaded to the system from the plans of previously treated patients.

Our study evaluates the resultant doses to the parotid glands for oropharyngeal radiotherapy planning through the use of the RP software, comparing two different knowledge‐based Oropharynx models. The first model, known as the ‘separated model’, contains parotid dose data from previously treated patients separated into contralateral and ipsilateral, while the second model, known as the ‘combined model’, combines the parotid data as one structure called ‘parotids’. Fogliata et al^17^ also investigated this comparison in a recent study and found that there was little difference between the two models for the resultant parotid dose. This current study sought to further validate this technique.

## Methods

### Patient selection for RP model creation

A RP model requires a library to be created from the treatment plans of previously treated patients. Patients used in this study were obtained from the ethics approved head and neck database (NSLHD reference: RESP/15/255) and had undergone definitive radiotherapy for T1‐4, N0‐3 oropharyngeal SCC. A cohort of seventy patients was uploaded to the model, with the primary tumour locations including tonsil (*n* = 38), base of tongue (*n* = 29) and pharynx (*n* = 3).

Patients had been treated using a standard departmental protocol consisting of a simultaneous integrated boost (SIB) with a 6 MV dual arc VMAT technique. The prescription dose was 70 Gy in 35 fractions to the high‐risk volume, 63 Gy in 35 fractions to the intermediate‐risk volume and 56 Gy in 35 fractions to the prophylactic lymph node volume.

The higher dosed overlapping planning target volumes (PTV) were removed from the lower dosed PTVs creating cropped PTVs (cPTV), making it easier to assess the outliers using the RP model configuration statistics.

### RP model configuration

The plans and matched contours of the 70 patients were exported from the Eclipse planning system (version 13.6) to RP model configuration for subsequent training. These data were used to train the model, generating mathematical parameters through the analysis of the geometric and dosimetric statistics of the 70 uploaded patients plans and contours.

The separated model was created first. The OARs trained for this model included the ipsilateral parotid, contralateral parotid, larynx, spinal cord, spinal cord planning risk volume (PRV), brainstem and mandible. Determining the ipsilateral parotid was a manual process and was considered to be the side the high‐risk volume favoured. The opposite parotid was trained as contralateral parotid.

### Model verification using model analytics

The Oropharynx model was then exported to Model Analytics (MA), a cloud‐based Varian endorsed programme on the MyVarian website, providing statistics of potential outlying plans, contours and dose volume histograms (DVH's) that would potentially decrease the integrity of the model. The results from the programme were assessed by reviewing each contour that the report highlighted in the clinical plan and removing it from the model if it was deemed to be an outlier that would degrade the quality of the model.

The RP statistics were then assessed to view any potential outliers missed in the previous steps. Again, any contours that were highlighted as outliers in the RP statistics were viewed in the plan and removed from the model if they were deemed to be an outlier.

Upper, lower and line objectives and priorities were then created in model configuration for each target and OAR that aimed to achieve the standard departmental protocol objectives. The cPTV's were used for the optimisation process. The gross tumour volume (GTV) structure (GTV Primary volume/GTV Primary Volume + GTV Nodal Volume) was also removed from cPTV high dose (cPTV HD) creating optimising PTV HD (oPTV HD). The GTV and oPTV HD were attached to the PTV HD objectives and priorities for the DVH estimation process. After the optimising values had been generated, optimisation objectives (see Table 1) were added to these structures manually to ensure adequate coverage.

**Table 1:** Manual optimisation objectives.

Structure	Type	Dose (%)	Priority
GTV	Upper	102.5	80
GTV	Lower	102	100
CTV HD	Lower	101	80
CTV ID	Lower	102	80
CTV LD	Lower	102	80

CTV = clinical target volume; GTV = gross tumour volume; HD = high dose; ID = intermediate dose; LD = low dose.

For the serial organs or PRVs of these structures, where maximum constraints were the only constraint in the departmental protocol, a fixed upper objective and priority was used. For the larynx and parotids, where the accepted dose was more influenced by geometric factors, a generated line objective and priority was added. Tuning structures such as shoulders were contoured, and optimising objectives were attached as previously done before RP was introduced to our centre (see Table 2 for an overview of optimising values).

**Table 2:** Model optimisation objectives.

Target/Organ	Objective	Volume (%)	Dose (cGy or % of the specific target prescription)	Priority
PTV HD	Upper	0	101.7	100
	Lower	100	100.7	100
PTV ID	Upper	0	102.4	100
	Lower	100	100.8	100
PTV LD	Upper	0	102.7	100
	Lower	100	100.9	100
GTV	Upper	0	101.7	100
	Lower	100	100.7	100
Larynx	Line	Generated	Generated	Generated
Mandible	Upper	0	6850	80
Contralateral parotid	Line	Generated	Generated	Generated
Ipsilateral parotid	Line	Generated	Generated	Generated
Spinal cord	Upper	0	3500	75
Spinal cord PRV	Upper	0	3800	75
Shoulders	Upper	0	500	50

GTV = gross tumour volume; HD = high dose; ID = intermediate dose; LD = low dose; PTV = planning target volume.

**Figure 1:**
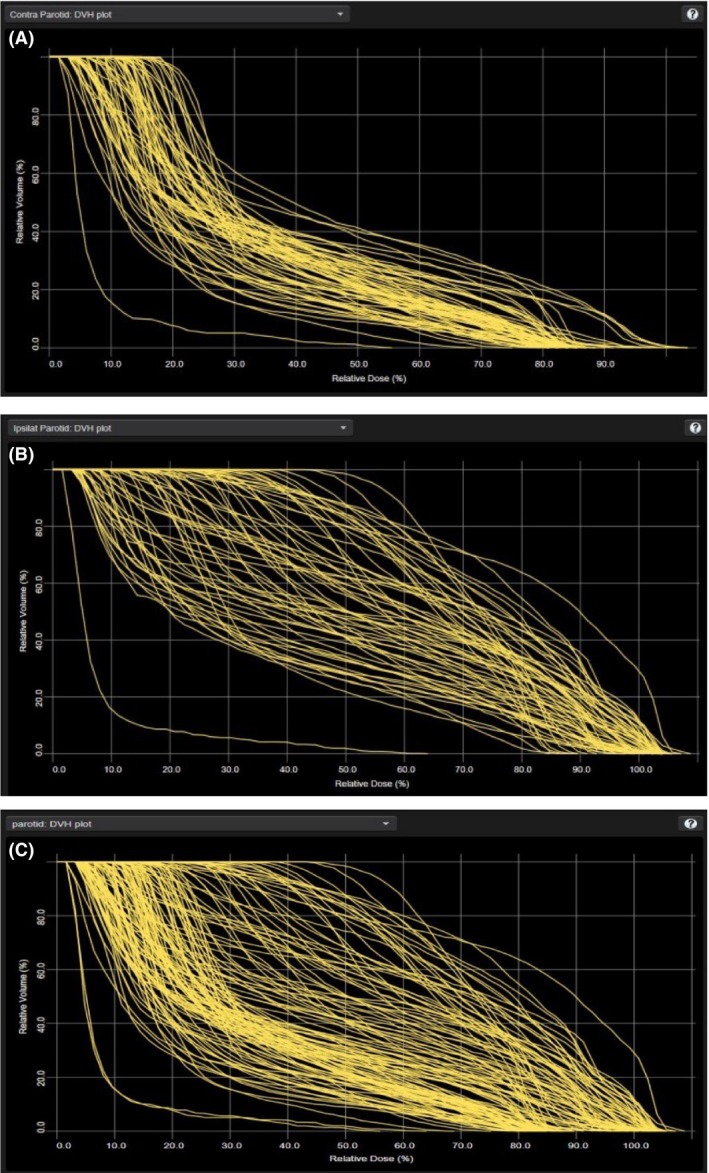
(A) The DVH data exported to the separated model for the ipsilateral parotid. (B) The DVH contralateral parotid data exported to the separated model. (C) The DVH parotid data for the combined model, a combination of the ipsilateral and contralateral DVH data from the separated model.

To create the two different models, the separated model was then duplicated. For each plan in the combined model, the parotids were adjusted from contralateral and ipsilateral, to one title called ‘Parotids’, which combined these data. The data were extracted again, and the model retrained with a generated line objective priority to be created for this one parotid structure for the optimising process. Figure 1A–C are examples of the DVH data used by the model to estimate the parotids for both models and shows the difference in the DVH statistics uploaded for the parotids in each model.

### Model validation

To validate and compare the two models, a cohort of 20 oropharynx patients not used in the creation of the library was optimised once using both models. The templated manual optimising objectives were added after the models had been attached but before commencing the optimising process. There was no more human interaction after the commencement of the optimisation. The resulting parotid mean and V50 doses were compared as these are the parameters assessed in the clinical setting. PTV coverage was also assessed to determine whether the models were producing clinically treatable plans.

## Results

During the validation process, the contours of 16 structures (10 larynxes, 5 ipsilateral parotids and 1 contralateral parotid) were removed as they were deemed to degrade the quality of the model. No whole plans were removed during the MA and RP statistic processes. The contours included in the model and the number of each OAR contour that remained after the validation process are presented in Table 3.

**Table 3:** Number of contour structures within each of the RP models.

Combined model		Separated model	
Contour	Number in model	Contour	Number in model
PTV HD	70	PTV HD	70
PTV ID	70	PTV ID	70
PTV LD	70	PTV LD	70
GTV	70	GTV	70
Parotid	134	Ipsilateral parotid	65
		Contralateral parotid	69
Spinal cord	70	Spinal cord	70
Spinal cord PRV	70	Spinal cord PRV	70
Larynx	60	Larynx	60
Brainstem	45	Brainstem	45
Mandible	40	Mandible	40

GTVp = gross tumour volume primary; HD = high dose; ID = intermediate dose; LD = low dose; PTV = planning target volume.

*Plan comparison:* Plans were produced successfully using both models on all 20 validation patients. Table 4 contains the characteristics of all 20 validation patients. Both models achieved departmental protocol for minimum PTV coverage for all 20 validation patients which were 95% of the prescribed dose covering 95% of the PTV volume. Table 5 gives an overview of the doses the OARs received from the two models.

**Table 4:** Patient characteristics.

Patient	ICD‐O site	Staging	Laterality	GTV vol (cc)	CTV vol (cc)
1	Tonsil	T3N2b	Left	6.9	51.7
2	Base of tongue	T4aN2c	Right	6.3	28.6
3	Base of tongue	T2N1	Right	2.1	29.0
4	Base of tongue	T2N2a	Right	12.7	93.0
5	Tonsil	T3N2c	Right	12.2	82.5
6	Base of tongue	T1N2b	Right	2.6	94.8
7	Base of tongue	T1N2b	Right	3.3	27.8
8	Tonsil	T4bN3	Left	2.4	189.8
9	Base of tongue	T1N1	Left	3.0	35.0
10	Tonsil	T1N2a	Left	2.2	55.7
11	Base of tongue	T2N2b	Right	4.9	39.6
12	Tonsil	T3N2c	Left	14.3	65.3
13	Base of tongue	T3N2b	Right	28.5	68.8
14	Base of tongue	T3N2a	Right	31.7	86.8
15	Tonsil	T4aN1	Right	20.2	53.8
16	Base of tongue	T1N2b	Right	22.1	99.2
17	Base of tongue	T1N3	Left	1.9	146.0
18	Base of tongue	T3N2b	Right	20.3	56.4
19	Tonsil	T1N3	Left	9.0	104.9
20	Tonsil	T2N0	Right	14.0	42.5

**Table 5:** OAR doses (Gy).

OAR	Model	Dose range	Mean dose	V50	Max (avg.)
Ipsi parotid	Combined	0–75.3	33.8	32.4	70.5
	Separated	0–74.5	35.5	34.2	70.2
Contra parotid	Combined	1.8–74.6	19.8	15.7	59.2
	Separated	1.9–75.0	20.5	15.4	59.7
Spinal cord	Combined	0.1–43.9	27.5	34.2	41.9
	Separated	0.1–44.3	27.5	34.0	42.1
Larynx	Combined	13.8–74.2	33.9	32.9	57.1
	Separated	14.4–71.2	33.9	32.9	57.3

**Figure 2:**
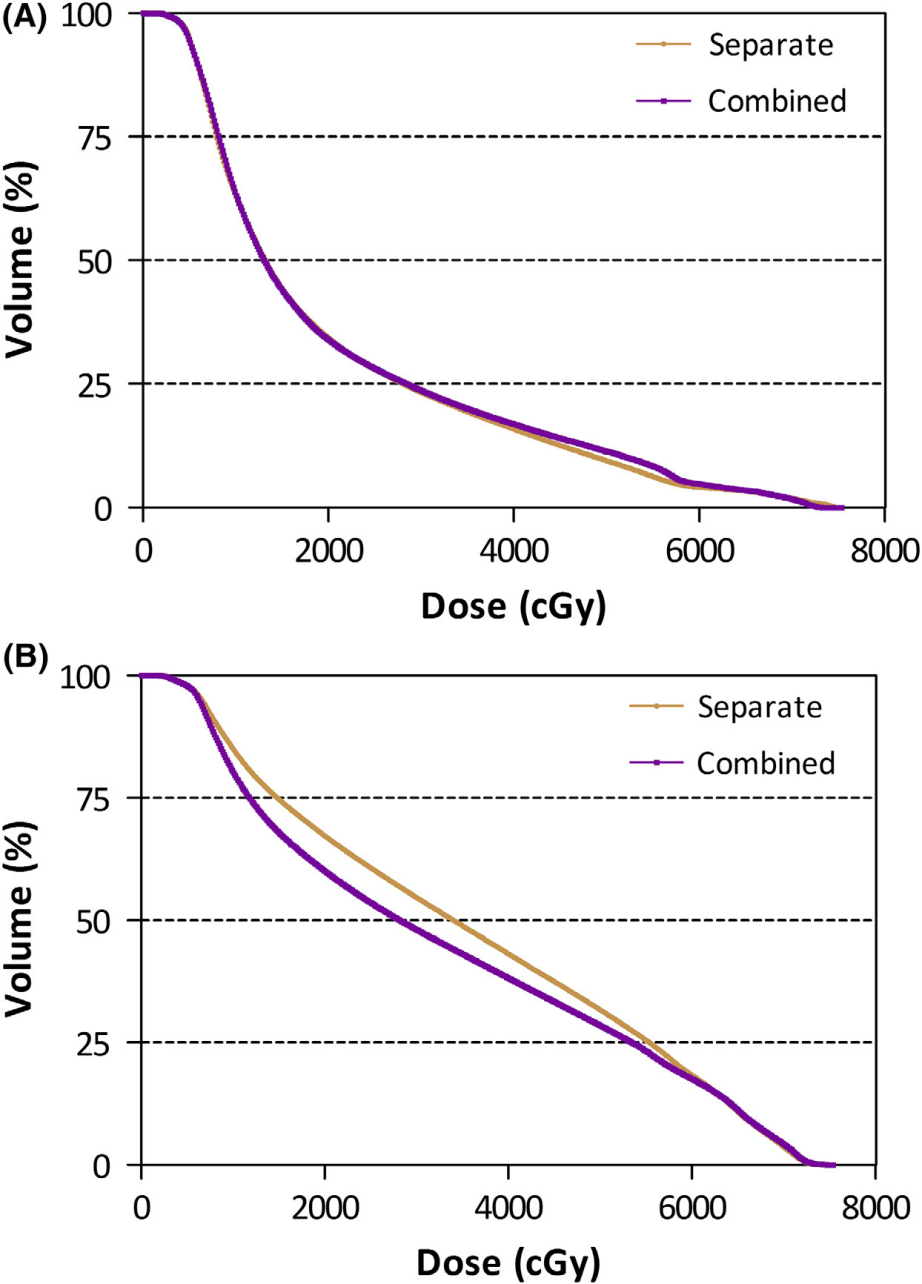
(A) Average contralateral parotid DVH for each model. (B) Average ipsilateral parotid DVH for each model.

Average DVH curves comparing the two models for contralateral and ipsilateral parotid doses are shown in Figures 2A and B. The combined model and separated model produced very similar contralateral parotid doses, but a lower ipsilateral parotid mean dose was seen in 16 of the 20 cases (average difference 1.8 Gy) and lower contralateral parotid doses in 11 of the 20 cases.

## Discussion

With excellent survival outcomes in patients with oropharyngeal cancers,^3^, ^4^ improved radiation treatment techniques are important to prevent long‐term treatment‐related toxicity. Using Varian's KBP software RP, two models using 70 previously treated patients with Oropharyngeal SCC were developed. Results from our study indicate no benefit for the contralateral parotid in separating parotid data into ipsilateral and contralateral when constructing models, with a potential improvement in ipsilateral doses. This is similar to results from Fogliata et al^17^ who also found little difference in the parotid doses between models. There were also no significant trade‐offs between the results of the OAR's produced by both models which can be seen in Table 5.

In our centre, planning staff have historically placed less importance on the optimisation of the ipsilateral parotid, with the belief that once the dose exceeds tolerance, there is no substantial benefit in small improvements in dose. Zhang et al^18^ and Ren et al^19^ explain the importance of parotid sparing to negate the potential for shrinkage and medial migration of the glands during treatment increasing dose. Keeping the dose to the parotids as low as possible has been shown to have a direct impact on the development of xerostomia, functional recovery of the glands and quality of life. By including the optimised contralateral parotid DVH data in the model, RP has increased quality data to use for the optimisation process which explains the improved ipsilateral doses.

The results of the study reinforce the notion that it is important to have quality plans within a model for it to utilise when estimating optimisation values for structures. Optimal plans used in models should have a positive influence on the results of RP models as was seen with the ipsilateral parotid results.

This concept may have ramifications for other RP models and may help in designing model construction. The concept of combining duplicate structures in models could be explored for other organs depending on tumour location, with the potential for similar outcomes. Examples of such organs are the hippocampus for models designed for central nervous system (CNS) treatment and kidneys for treatments in the abdomen.

It is also an easier and neater process for model creation when data for duplicate structures are involved. Having a single structure that corresponds to both parotids will result in less user error when creating a model or during the optimising of a plan where structures are attached to their corresponding structure in the model.

## Conclusion

Separation of the parotids into ipsilateral and contralateral in models, matching the goals of planning, did not improve parotid results. Combining the parotids resulted in lower estimations for the ipsilateral parotid and therefore lower doses. The outcomes may influence the designing of similar models with duplicate structures.

## Conflict of Interest

The authors declare no conflict of interest.
